# Viscoelastic friction in sliding a non-cylindrical asperity

**DOI:** 10.1140/epje/s10189-025-00484-5

**Published:** 2025-04-29

**Authors:** M. Ciavarella, M. Tricarico, A. Papangelo

**Affiliations:** Department of Mechanics Mathematics and Management, TriboDynamics Lab, Politecnico di Bari, Via Orabona 4, 70125 Bari, Italy

## Abstract

**Abstract:**

We investigate the 2D contact problem of sliding a non-cylindrical punch on a viscoelastic halfplane, assuming a power law shape $\left| x\right| ^{k}$ with $k>2$. We find with a full boundary element numerical solution that the Persson analytical solution for friction, which works well for the cylindrical punch case assuming the pressure remains identical in form to the elastic case, in this case leads to significant qualitative errors. However, we find that the friction coefficient follows a much simpler trend; namely, we can use as a first approximation the solution for the cylinder, provided we normalize friction coefficient with the modulus and mean pressure at zero speed, despite that we show the complex behaviour of the pressure distribution in the viscoelastic regime. We are unable to numerically solve satisfactorily the ill-defined limit of sharp flat punch, for which Persson’s solution predicts finite friction even at zero speed.

**Graphic Abstract:**

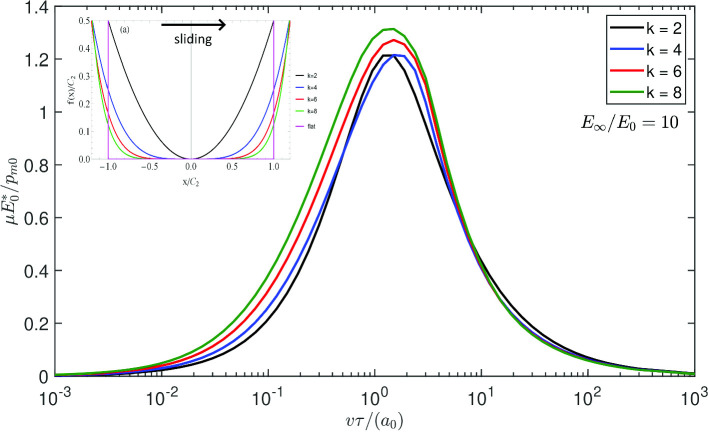

## Introduction

Friction in rubber-like materials is a topic of huge importance largely due to hysteretic losses. Obvious application range from tires in vehicles to seals and robotics [1–16]. A classical configuration for macroscopic object is the sphere or the cylinder sliding or rolling on a flat rubber substrate for which various researchers have conducted experimental investigations [17–20]. When lubrication forms a sufficiently large film, roughness is not important and adhesive effects are removed, and the idealization of the smooth sphere or cylinder is appropriate. Moreover, the two conditions of sliding and rolling become identical. For a single relaxation time, Hunter [21] produced a complex exact result for the rolling friction, but Persson [22] produced a simplified solution which is valid for arbitrary relaxation spectra, and is also simple because it assumes for the pressure distribution, the same pressure distribution under elastic contact.

**Fig. 1:**
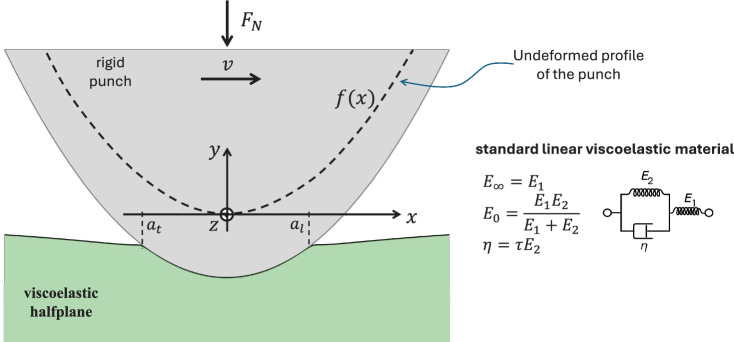
Schematic of the 2D power law profile sliding over a linear viscoelastic halfplane. We assume the punch has thickness *L* in the *z* direction. A schematic for a standard linear viscoelastic material is shown in the Kelvin representation where $\{E_1,E_2\}$ have the dimension of an elastic modulus, $\eta$ is the viscosity and $\tau$ is the characteristic time of the material. $\{a_l,a_t\}$ are the contact semi-widths, respectively, at the leading and trailing edge. In the elastic limit (very low or very high sliding velocity) $|a_l|=|a_t|$

In the case of rolling, the cylindrical or spherical geometries are obviously the only one of interest. However, in the case of sliding, any shape is possible. In particular, it may be of interest to find some “optimal shape” for minimizing or maximizing friction, depending on application. In these respects, the present paper investigates the effect of shape of a 2D asperity on the viscoelastic friction (Fig. 1).

In steady-state conditions, the friction force $F_{R}$ is given by the ratio of the energy loss per unit time $\overset{\cdot }{W}$ to the sliding velocity *v*

1 $$\begin{aligned} F_{R}=\frac{\overset{\cdot }{W}}{v} \end{aligned}$$

but if the pressure *p* and the vertical displacements *u* fields are known (in particular, the displacement within the contact area is known for a rigid punch), the frictional force per unit length is also given by the integral

2 $$\begin{aligned} f_{R}=\frac{F_{R}}{L}=-\int _{area}p\left( x\right) \frac{du}{dx}dx \end{aligned}$$

where *u* is the vertical displacement and *x* the coordinate along the sliding direction (we have assumed a plane contact problem of width *L* in the z direction, see Fig. 1), and the integral is extended over the contact area segment. Notice that the second formulation (Eq. 2) seems to imply zero friction for the limit case of a flat punch, and this curious result was one of the original motivations of the present paper, since we do not see how a flat punch would lead to no dissipation—in other words, it seemed the two methods would lead to a different result. However, we shall observe that in linear elasticity/viscoelasticity, a perfectly flat punch would imply edge singularities in the pressure distribution. Consequently, the substrate slope at the contact edges $x=\pm a$ is ill-defined, being 0 within the contact and $\pm \infty$ just outside the contact. A possible approach would be to tackle the problem considering a flat punch with rounded edges and then taking the limiting process where the radius of curvature of the rounds vanishes. This process leads to similar numerical difficulties as the present effort with power law punch and is therefore not attempted (refer to Ref. [23] for a detailed analysis of the contact problem of a flat punch with rounded edges). The limit of flat punch remains ill defined for our understanding as we have tried to reach convergence but failed with our numerical approach. Contrary, we did not find convergence problems for power law profiles $\propto |x|^k$ with exponent up to $k=8$.

Notice that the integral expression in (Eq. 2) and the energy approach of (Eq. 1) are equivalent in computing $F_{R}$. Persson [22] started from (Eq. 1) and introduced the assumption that the pressure distribution in the viscoelastic problem preserves the shape of the elastic one, which, for the case of the cylinder, was a good approximation. The first method (Eq. 1) identifies a time-dependent change of the deformation state of the elastomer and, therefore, energy loss. It was used by Persson [22] showing that the energy dissipated $\Delta E_{diss}$ during the time period $t_{0}$ equals the work done by the stresses $\mathbf {\sigma }$ on the displacements and hence

3 $$\begin{aligned} \Delta E_{diss}=F_{R}vt_{0}=\int _{area}d^{2}xdt\overset{\cdot }{\textbf{u} }\cdot \mathbf {\sigma } \end{aligned}$$

Using 2D Fourier transforms [22] obtains eventually that

4 $$\begin{aligned} F_{R}=2\frac{\left( 2\pi \right) ^{2}}{v}\int d^{2}q\frac{\omega }{q}\operatorname {Im}\frac{1}{E^{*}\left( \omega \right) }\left| p\left( \textbf{q}\right) \right| ^{2} \end{aligned}$$

where $\mathbf {q=}\left( q_{x},q_{y}\right)$ is a 2D vector, and $E^{*}\left( \omega \right) =E\left( \omega \right) /\left( 1-\nu ^{2}\right)$ is the plain strain viscoelastic modulus at circular frequency $\omega$ ($\nu$ is the Poisson ratio assumed independent on the frequency of excitation and *E* is the Young modulus). It is clear that the integral, having only positive values as integrand, needs to be nonzero. This solution takes into account the stiffening of the viscoelastic modulus with frequency and hence the reduced contact area at high speed.

To make an example on the importance of the pressure distribution, consider the simplest case of a single constant pressure element $p\left( x\right) =p$ on $\left( -a,a\right)$. Using Eq. 2, the friction force per unit length would be

5 $$\begin{aligned} f_{R}=-p\left( u\left( a\right) -u\left( -a\right) \right) \end{aligned}$$

This results in a bell-shaped curve for friction coefficient $\mu =\frac{f_{R} }{f_{N}}=\frac{f_{R}}{2ap}=-\frac{u\left( a\right) -u\left( -a\right) }{2a}$ which can be found very easily with analytical results in Afferrante and Carbone [24], and turns out to have a maximum

6 $$\begin{aligned} \mu _{\max }\frac{E_{0}^{*}}{p}\simeq 0.67 \end{aligned}$$

for standard solid with $E_{0}^{*}$ as relaxed modulus (the modulus at zero frequency) for a speed $\frac{v\tau }{a}$ just above 1. More precisely, the maximum varies in the range between 0.645 and 0.71 changing the ratio of the instantaneous to relaxed viscoelastic modulus $E_{\infty }/E_{0}$ from 10 to 1000. For cylinder, we have from Persson [22] values reported as $\mu _{\max }R/a_{0}$ where *R* is radius of the indenter and $a_{0}$ the contact semi-width at zero velocity which for the elastic Hertz theory is $a_{0} =\sqrt{\frac{4f_{N}R}{\pi E_{0}^{*}}}$, but here we prefer to report them to the mean pressure at zero speed $p_{m0}=\frac{f_{N}}{2a_{0}}$ and we find from Persson [22] solution

7 $$\begin{aligned} \mu _{\max }\frac{E_{0}^{*}}{p_{m0}}=1.25-1.472 \end{aligned}$$

for the range $E_{\infty }/E_{0}=10-1000$ (which is of interest for many viscoelastic materials [25, 26]), so about double the value for uniform pressure (6).

The friction coefficient for a power law punch is easy to compute extending Persson’s solution [22]. It will be shown that the shape of the indenter leads to important qualitative effects. Therefore, Persson’s result will be compared with a full numerical solution which is based on the second method (Eq. 2), after solving numerically the contact problem.

The numerical code is based on the boundary element method. The contact domain is discretized in *M* equally spaced elements corresponding to *M* interface nodes located in the middle of each element. The contact pressure is assumed to be constant on each element and steady travelling at the sliding velocity *v*, for which the nodal deflection can be computed by using the fundamental solution derived for a standard linear viscoelastic material in Ref. [27]. The contact is detected by means of a penality method [28]: a stiff contact stiffness is introduced at the interface so that a small interpenetration between the punch and the substrate is allowed. Given the normal force, the punch profile and the sliding velocity the nonlinear contact problem is solved by a Newton–Raphson scheme using the $\hbox {MATLAB}^{\copyright }$ built-in function *fsolve*. More details are provided in references [29, 30].

## A power law punch

**Fig. 2:**
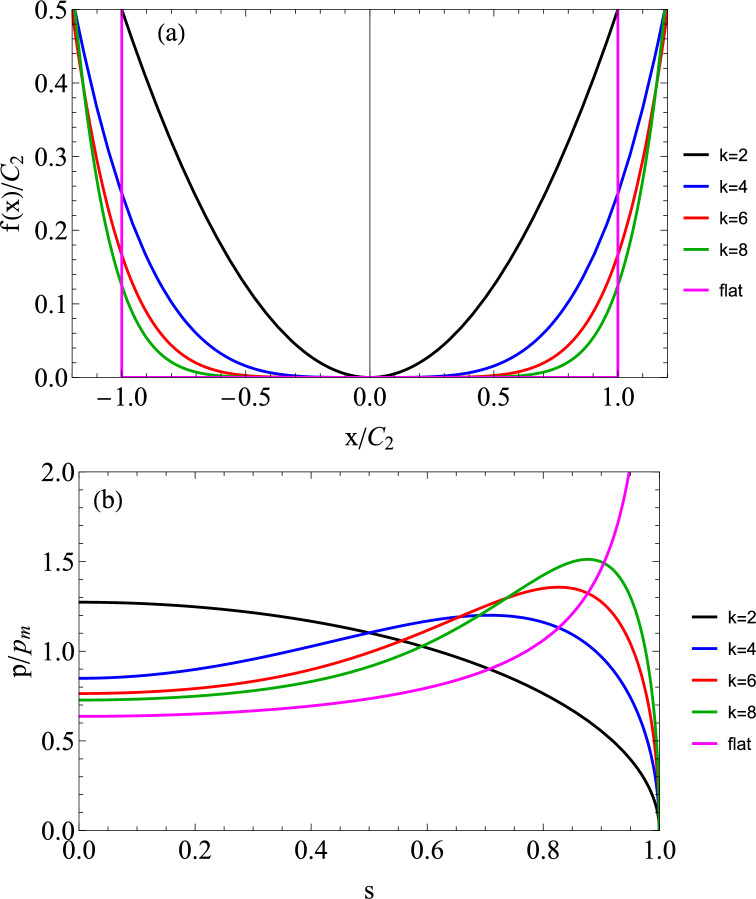
**a** Profiles of the power law punches considered in this study $f(x)=\frac{\left| x\right| ^{k}}{kC_{2}^{k-1}}$ and of a flat punch, shown in dimensionless form. **b** The dimensionless (elastic) pressure distribution $\frac{p\left( s\right) }{p_{m}}=\frac{p_{0}\left( s\right) }{p_{m0}}$ as in Eq.(12) as function of the dimensionless coordinate $s=x/a$ for power law punch for $k=2,4,6,8$ and the limit flat punch solution, respectively, black, blue, red, green, and purple curves. Notice that this pressure also holds for viscoelastic case for very low speed

Let us therefore consider a 2D punch with power law profile $f\left( x\right) =C_{1}\left| x\right| ^{k}=\frac{\left| x\right| ^{k}}{kC_{2}^{k-1}}$, where $C_{2}$ has the dimension of a length (see Fig. 2a). For an Hertzian punch obviously $k=2$ and $C_{2}=R$. The elastic pressure is (for even *k*)

8 $$\begin{aligned} p\left( s\right) =-\frac{E^{*}C_{1}k}{2\sqrt{\pi }}a^{k-1}r\left( s,k\right) =-\frac{E^{*}}{2\sqrt{\pi }}\left( \frac{a}{C_{2}}\right) ^{k-1}r\left( s,k\right) \end{aligned}$$

where $$s=x/a$$, $$r\left( s,k\right) =\frac{\Gamma \left( \frac{k-1}{2}\right) }{\Gamma \left( \frac{k}{2}\right) }\sqrt{1-s^{2}}_{2} F_{1}\left( 1,1-\frac{k}{2};\right. \left. \frac{3-k}{2};s^{2}\right) $$, where $$\Gamma $$ is the Gamma function and $$ _{2}F_{1}$$ is the standard hypergeometric function. The normal load per unit thickness is

9 $$\begin{aligned} f_{N}=C_{1}kE^{*}a^{k}\frac{\sqrt{\pi }}{2}\frac{\Gamma \left( \frac{k+1}{2}\right) }{\Gamma \left( \frac{k}{2}+1\right) } \end{aligned}$$

and mean pressure is obviously $p_{m}=\frac{f_{N}}{2a}$. This solution is correct for the limit cases of vanishing sliding speed also for the viscoelastic problem using $E^{*}=E_{0}^{*}$ and being $p_{m0} =f_{N}/2a_{0}$ one obtains^1^

11 $$\begin{aligned} \frac{p_{m0}}{E_{0}^{*}}= &   \frac{\sqrt{\pi }}{4}\frac{\Gamma \left( \frac{1+k}{2}\right) }{\Gamma \left( 1+\frac{k}{2}\right) }\nonumber \\  &   \left[ \left( \frac{4}{\sqrt{\pi }}\right) ^{1/k}\left( \frac{f_{N}}{2C_{2}E_{0}^{*} }\frac{\Gamma \left( 1+\frac{k}{2}\right) }{\Gamma \left( \frac{1+k}{2}\right) }\right) ^{1/k}\right] ^{k-1} \end{aligned}$$

Also, we can write

12 $$\begin{aligned} \frac{p_{0}\left( s\right) }{p_{m0}}=-\frac{2}{\pi }\frac{\Gamma \left( \frac{k}{2}+1\right) }{\Gamma \left( \frac{k+1}{2}\right) }r\left( s,k\right) \end{aligned}$$

which is plotted in Fig. 2b as $\frac{p\left( s\right) }{p_{m}}=\frac{p_{0}\left( s\right) }{p_{m0}}$ for an elastic case, showing slowly the tendency towards the flat punch solution $p_{0}\left( s\right) =\frac{f_{N} }{\pi a_{0}}\frac{1}{\sqrt{1-s^{2}}}=\frac{2p_{m0}}{\pi }\frac{1}{\sqrt{1-s^{2}}}$.

From Eq. (9) and using $C_{1}=\frac{1}{kC_{2}^{k-1}}$, the contact semi-width at zero speed is

13 $$\begin{aligned} \frac{a_{0}}{C_{2}}=\left( \frac{f_{N}}{\frac{\sqrt{\pi }}{2}\frac{\Gamma \left( \frac{k+1}{2}\right) }{\Gamma \left( \frac{k}{2}+1\right) }C_{2}E_{0}^{*}}\right) ^{1/k} \end{aligned}$$

**Fig. 3:**
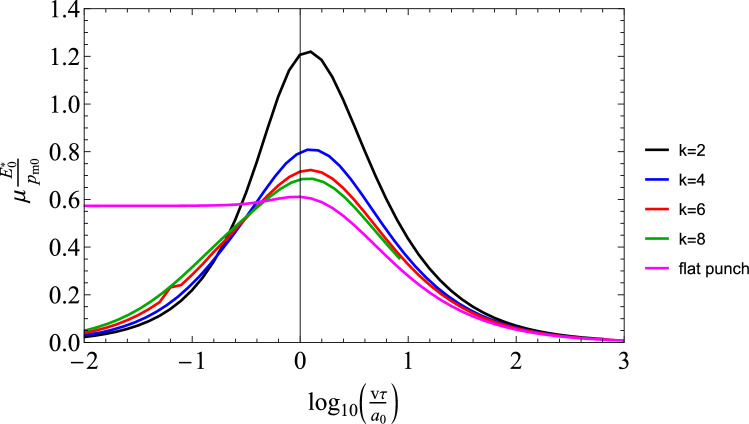
Using Persson’s solution [22] for a power law punch in terms of $\mu E_{0}^{*}/p_{m0}$ as a function of the dimensionless velocity $v\tau /a_{0},$ assuming “elastic” pressure distribution, for $k=\left[ 2,4,6,8\right]$ and the limit flat punch solution, respectively, black, blue, red, green, and purple curves

**Fig. 4:**
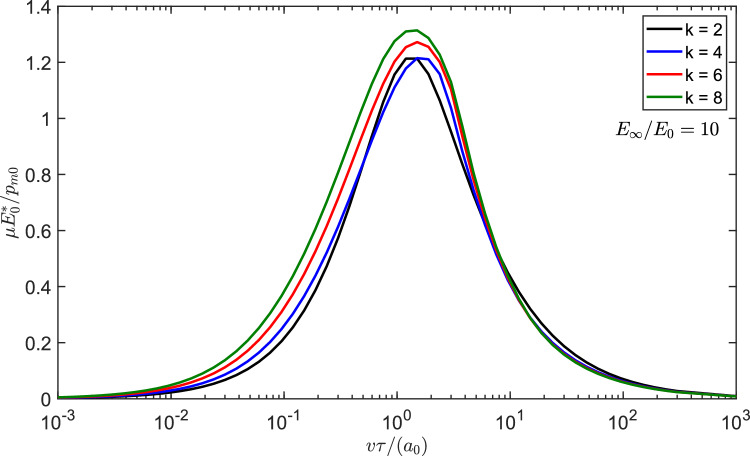
BEM solution for $\mu E_{0}^{*}/p_{m0}$ for power law punch for $k=\left[ 2,4,6,8\right]$

**Fig. 5:**
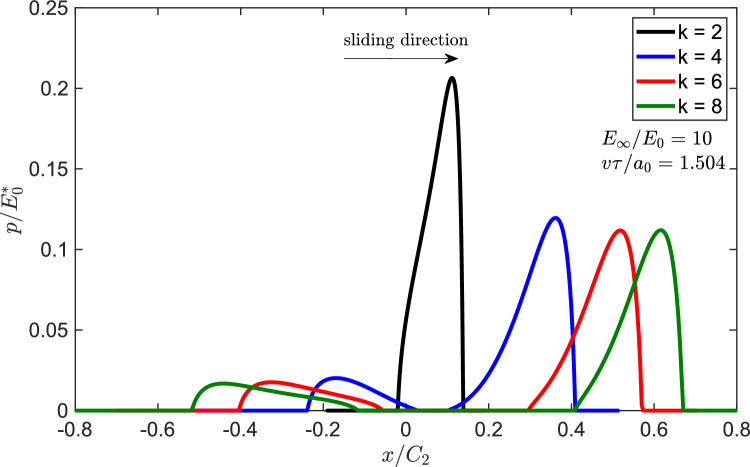
The pressure distribution obtained from BEM solution for $v\tau /a_{0}=1.504$ for power law punch for $k=\left[ 2,4,6,8\right]$

**Fig. 6:**
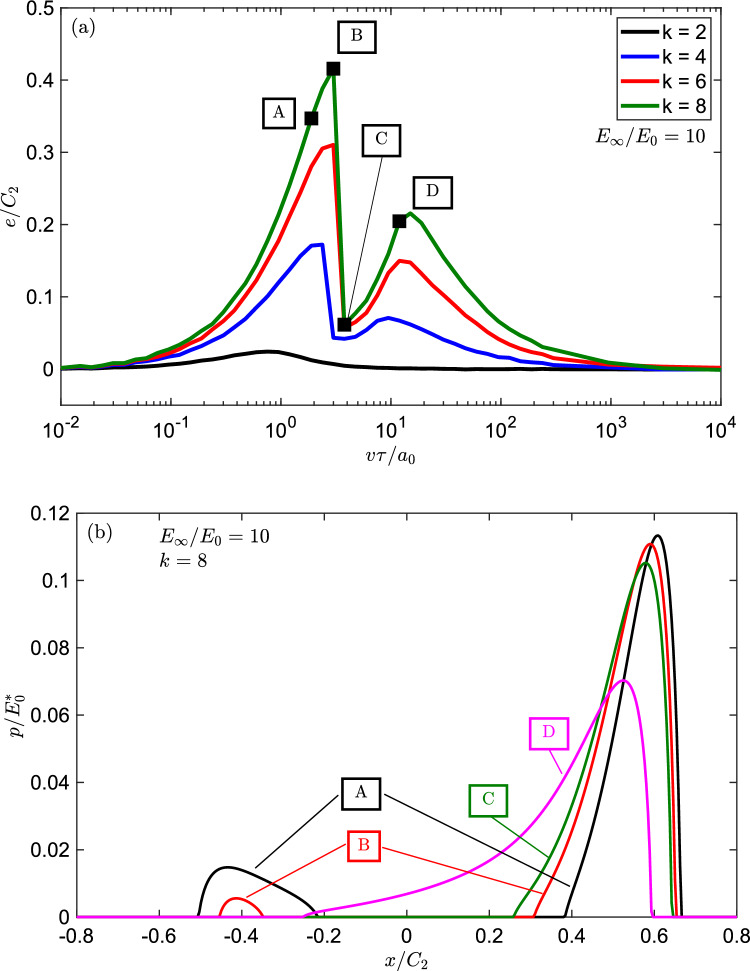
**a** The eccentricity of the pressure distribution $e/C_{2}$ calculated with Eq. (16) using the pressure distribution obtained numerically with the BEM code as function of $v\tau /a_{0}$ for power law punch for $k=\left[ 2,4,6,8\right]$. **b** The pressure distributions for the sliding velocities $v\tau /a_{0}=\left[ 1.89, 3.00, 3.78, 11.94\right]$ and $k=8$ (labelled (A, B, C, D)) are shown

**Fig. 7:**
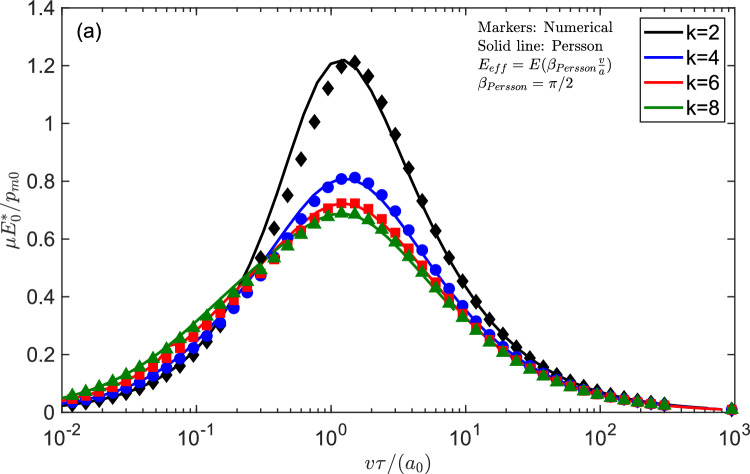
$\mu E_{0}^{*}/p_{m0}$ as a function of the dimensionless velocity $v\tau /a_{0}$ for Persson’s solution [22] (solid lines) compared with the curves that can be obtained numerically assuming the pressure distribution remains symmetrical and “elastic” but using an effective modulus $E_{eff}^{*}\left( \beta _{Persson}\frac{v}{a}\right)$ that satisfies Eq. (17) $\left( \beta _{Persson}\simeq \pi /2\right)$

For a power law indenter, Persson’s viscoelastic friction theory leads to (refer to Appendix)

14 $$\begin{aligned} \mu= &   \frac{f_{R}}{f_{N}}=\frac{8}{\pi ^{3}}f_{N}\left( \frac{\Gamma \left( \frac{k}{2}+1\right) }{\Gamma \left( \frac{k+1}{2}\right) }\right) ^{2}\nonumber \\  &   \int _{0}^{\infty }dq_{x}\operatorname {Im}\frac{1}{E_{eff}\left( q_{x}v\right) }I\left( q_{x}a,k\right) ^{2} \end{aligned}$$

where $\Gamma \left( x\right)$ is the Gamma function, $q_{x}$ is the wavenumber, $E_{eff}\left( q_{x}v\right)$ is the effective modulus at the frequency $\left( q_{x}v\right)$ and $I\left( q_{x}a,k\right) =\int _{0}^{1}ds\cos \left( q_{x}as\right) r\left( s,k\right)$ is an integral function depending on the punch shape. The integrals require numerical integration, except in the limit of a flat punch and a standard material, for which we get a closed form result

15 $$\begin{aligned} \mu =\frac{2f_{N}}{\pi a}\left( \frac{1-E_{0}/E_{\infty }}{E_{0}^{*} }\right) \frac{I_{0}\left( \frac{a}{\tau v}\right) K_{0}\left( \frac{a}{\tau v}\right) }{\tau v/a} \end{aligned}$$

where $I_{0}\left( \frac{a}{\tau v}\right)$ and $K_{0}\left( \frac{a}{\tau v}\right)$ are, respectively, the Bessel functions of the first and second kind and the contact area is fixed, i.e. $a=a_{0}$. Figure 3 shows the Persson’s results in terms of $\mu E_{0}^{*}/p_{m0}$ for $k=\left[ 2,4,6,8\right]$ and for the limiting case of a flat punch for a standard linear solid with $E_{\infty }/E_{0}=10$. It can be seen that maximum decreases with increasing *k* from the Hertzian case to values about 1/2 smaller, similarly to what we predicted in moving from the Hertz to the uniform pressure problem. This is reasonable since the higher *k* do look more “uniform” (as long as $k\le 8$, as above this value we find singularities at the edges, which according to Persson’s theory would lead to dramatic effects at low speeds). Hence, this is the result given the assumptions in Persson’s theory: (i) dissipation is computed for the “elastic” pressure distribution (ii) change of contact area is estimated with the change of modulus. But is this result realistic? In the next paragraph, we conduct a full numerical investigation.

## Numerical results

Given the quite strong assumption in Persson’s theory and mainly that dissipation is computed from the “elastic” pressure distribution, we use a full BEM formulation described above (see also Ref. [29, 30]), to obtain friction curves from Eq. 2. In particular, results are given as a function of speed in Fig. 4 as $\mu E_{0}^{*}/p_{m0}$ for $E_{\infty }/E_{0}=10$ (a standard linear viscoelastic material is considered throughout the paper).

It is clear that the maximum friction coefficient seems to depend much less on the shape of the punch *k* than what Persson’s theory predicted (compare Fig. 4 with Fig. 3), and this leads to a surprisingly simple result in itself. In order to explore more in depth what happens therefore for the case varying *k*, we plot the pressure distribution in Fig. 5 in a representative case corresponding to about the friction coefficient peak $v\tau /a_{0}\simeq 1.504$. For large *k* the contact segment is not compact and this contrasts with the approximation in Ref. [22] to use for the viscoelastic problem a pressure distribution that has the same shape of that in the elastic problem. Let us define the eccentricity *e* of the pressure distribution as follows:

16 $$\begin{aligned} e=\frac{1}{f_{N}}\int _{area}p\left( \zeta \right) \zeta d\zeta \end{aligned}$$

where $\zeta =x-a_{m}$ is thein-plane coordinate centred with respect to the contact segment where $a_{m}=\left( a_{t}+a_{l}\right) /2$ and $\left\{ a_{t},a_{l}\right\}$ are, respectively, the x-coordinates of the trailing and leading edge.

The eccentricity *e* is positive when the pressure distribution is skewed towards the leading edge, it vanishes for symmetrical pressure distribution and is negative when the pressure distribution is skewed towards the trailing edge. Inspection of Fig. 6a revels that the eccentricity for $k=\left[ 4,6,8\right]$ is much higher than the Hertzian case and increases with *k*. The curve for $k=2$ remains smooth over all the range of velocities investigated, while for $k=\left[ 4,6,8\right]$ a jump appears at $v\tau /a_{0}\approx 3$. This is related to the evolution of the contact patch with the sliding velocity. The pressure distributions for the sliding velocities $v\tau /a_{0}=\left[ 1.89,3.00,3.78,11.94\right]$ and $k=8$ (labelled (A, B, C, D) in Fig. 6) are shown in Fig. 6b. Notice that the jump in the measure of the eccentricity comes from a transition from a not compact contact area (cases (A, B)) where separation happens in the inner part of the contact to a compact contact area clustered at the leading edge (cases (C, D)).

The eccentricity “*e*” shown in in Fig. 6a represents the moment arm that multiplied by the normal force gives the moment introduced by the pressure distribution. In this respect the pressure distribution labelled as (B) has a large eccentricity as it shows two “spikes” both at the leading and trailing edge, while the case labelled (C) has a relatively low eccentricity as the contact patch is compact and located towards the leading edge (see Fig. 6b). Figure 6b clarifies that Persson approximation of using the elastic pressure distribution to determine the frictional dissipation may be considered accurate only for the Hertzian geometry, while for $k>2$ the pressure distribution in the viscoelastic problem largely differs from that in the elastic problem.

Notice that, giving the steep power law profile used here (e.g. $k=8$), we checked the validity of the small-slope/small-deformations assumption of linear elasticity computing for the case $k=8$ the interface local slope $du_z/dx$ over all the range of velocity considered, being $u_z$ the substrate deflections. We found $\max (|du_z/dx|)\simeq 0.06\ll 1$ which supports the validity of linear elasticity.

To clarify better the spirit behind the theory derived by Persson, we also made a further investigation. We used the elastic symmetrical pressure distribution, sliding on the viscoelastic halfplane (i.e. not solving the full contact problem), but adjusting the contact area in order to consider an effective modulus $E_{eff}^{*}$ corresponding to the excitation frequency *v*/*a* so that the elastic solution at the considered effective modulus

17 $$\begin{aligned} \frac{a}{C_{2}}=\left( \frac{f_{N}}{\frac{\sqrt{\pi }}{2}\frac{\Gamma \left( \frac{k+1}{2}\right) }{\Gamma \left( \frac{k}{2}+1\right) }C_{2} E_{eff}^{*}\left( \beta _{Persson}\frac{v}{a}\right) }\right) ^{1/k} \end{aligned}$$

is always satisfied and $\beta _{Persson}$ is a corrective factor to match the solution proposed by Persson. In other words, we take an increase of the viscoelastic modulus due to increase of loading frequency. It is seen from Fig. 7 that using $\beta _{Persson}\simeq \pi /2$ fits in all cases Persson’s solution, which agrees with the idea that Persson solution corresponds to a halfpulse of size 2*a* of pressure travelling on the viscoelastic halfplane.

## Conclusions

We have investigated the 2D contact problem of a punch sliding on a viscoelastic halfplane, to assess the induced viscoelastic losses and hence friction for sliding in conditions where roughness and adhesion are negligible and hence do not contribute to friction. We found that the Persson’s approximate analytical solution, which works very well for the cylindrical punch assuming the shape of the pressure remains the elastic symmetrical one, does not seem to work for other cases, due to higher eccentricity. However, we found that friction seems to remain nearly independent on shape of the punch when considering the results normalized as $\mu E_{0}^{*}/p_{m0}$ which is a quantity which requires no numerical solution of the viscoelastic contact problem. In other words, it seems the cylindrical solution can be used also for other shapes, within the limits of our investigations (standard material, and $k=[2, 4,6, 8]$). We have not been able to obtain very accurate results for the limit case of a sharp punch, since the singularities in pressures at the square corners induce very hard numerical problems, and hence this case poses computational challenges and remains not well defined. The penality method used in our numerical simulations had implicitly introduced a regularization of the contact problem [23, 31] equivalent to an “effective round” of the sharp corners. Nevertheless, reducing the contact stiffness $k_c$ implied a stronger approximation of the contact solution and increasing $k_c$ lead to convergence problems; hence, the limit of the flat punch remained ill defined for our understanding. The results may be a starting point to optimize the shape of sliding indenters, for minimizing or maximizing friction, depending on the desired function.

## Data Availability

The dataset generated for this article is available on Zenodo at DOI: https://doi.org/10.5281/zenodo.15283745.

## 5 Appendix—Persson’s solution for power law punch

To apply Persson solution [22], we compute the Fourier transform of the pressure (12), namely $p\left( \varvec{q}\right) =\frac{1}{\left( 2\pi \right) ^{2}}\int d^{2}xp\left( x\right) e^{-i\varvec{q\cdot x}}$ giving

18 $$\begin{aligned} p\left( \varvec{q}\right) =\delta \left( q_{y}\right) \frac{1}{2\pi }2\frac{E^{*}C_{1}k}{2\sqrt{\pi }}a^{k}I\left( q_{x}a,k\right) \end{aligned}$$

where given the symmetry of the pressure, $I\left( q_{x}a,k\right) =\int _{0}^{1}ds\cos \left( q_{x}as\right) r\left( s,k\right)$. Therefore

$$\begin{aligned} p\left( \varvec{q}\right) =\delta \left( q_{y}\right) f_{N}\frac{1}{\pi ^{2}}\frac{\Gamma \left( \frac{k}{2}+1\right) }{\Gamma \left( \frac{k+1}{2}\right) }I\left( q_{x}a,k\right) \end{aligned}$$

For various even *k*, we have

$$\begin{aligned}&I\left( q_{x}a,k\right) =\pi ^{3/2}\frac{J_{1}\left( q_{x}a\right) }{2\left( q_{x}a\right) }\text { for }k=2\\&\quad =3\pi ^{3/2}\frac{\left( q_{x}a\right) J_{1}\left( q_{x}a\right) -2J_{2}\left( q_{x}a\right) }{4\left( q_{x}a\right) ^{2}}\text { for }k=4\\&\quad =-15\pi ^{3/2}\frac{\left( -8+\left( q_{x}a\right) ^{2}\right) J_{3}\left( q_{x}a\right) }{16\left( q_{x}a\right) ^{3}}\text { for }k=6\\&\quad =\frac{35\pi ^{3/2}\left( aq_{x}\left( 288-24a^{2}q_{x}^{2}+a^{4}q_{x} ^{4}\right) J_{1}\left( aq_{x}\right) -6\left( 192-24a^{2}q_{x}^{2} +a^{4}q_{x}^{4}\right) J_{2}\left( q_{x}a\right) \right) }{32\left( q_{x}a\right) ^{6}}\qquad \text { for }k=8 \end{aligned}$$

Then, using $\left| \delta \left( q_{y}\right) \right| ^{2} =\delta \left( q_{y}\right) \frac{L}{2\pi }$ where *L* is the width of the plane problem,

$$\begin{aligned} \left| p\left( \varvec{q}\right) \right| ^{2}=\delta \left( q_{y}\right) \frac{L}{2\pi }\frac{f_{N}^{2}}{\pi ^{4}}\left( \frac{\Gamma \left( \frac{k}{2}+1\right) }{\Gamma \left( \frac{k+1}{2}\right) }I\left( q_{x}a,k\right) \right) ^{2} \end{aligned}$$

We have that friction force (4), using $\omega =q_{x}v$

$$\begin{aligned} F_{R}= &   2\left( 2\pi \right) L\frac{f_{N}^{2}}{\pi ^{4}}\int d^{2} q\operatorname {Im}\frac{1}{E_{eff}\left( \omega \right) }\delta \left( q_{y}\right) \\  &   \left( \frac{\Gamma \left( \frac{k}{2}+1\right) }{\Gamma \left( \frac{k+1}{2}\right) }I\left( q_{x}a,k\right) \right) ^{2} \end{aligned}$$

or, changing the limits of integration and considering that $F_{R}$ is a real quantity, the friction coefficient $\mu$ *is*

19 $$\begin{aligned} \mu= &   \frac{F_{R}/L}{F_{N}/L}=\frac{f_{R}}{f_{N}}\nonumber \\= &   \frac{8}{\pi ^{3}}f_{N}\left( \frac{\Gamma \left( \frac{k}{2}+1\right) }{\Gamma \left( \frac{k+1}{2}\right) }\right) ^{2}\nonumber \\  &   \int _{0}^{\infty }dq_{x}\operatorname {Im}\frac{1}{E_{eff}\left( q_{x}v\right) }I\left( q_{x}a,k\right) ^{2} \end{aligned}$$

Notice, for a linear standard material

20 $$\begin{aligned} E_{eff}\left( \omega \right)&=\frac{E_{\infty }\left( 1-\varvec{i} \omega \tau \right) }{E_{\infty }/E_{0}-\varvec{i}\omega \tau }\end{aligned}$$

21 $$\begin{aligned} \left| E_{eff}\left( \omega \right) \right|&=E_{0}\sqrt{\frac{1+\left( \omega \tau \right) ^{2}}{1+\left( \frac{E_{0}}{E_{\infty } }\omega \tau \right) ^{2}}}\end{aligned}$$

22 $$\begin{aligned} \operatorname {Im}\frac{1}{E_{eff}}&=\left( \frac{1}{E_{0}}-\frac{1}{E_{1}}\right) \frac{\omega \tau }{1+\left( \omega \tau \right) ^{2}} \end{aligned}$$

## Footnotes

1 In the special case of cylindrical indenter
